# TIP aquaporins in *Cyperus esculentus*: genome-wide identification, expression profiles, subcellular localizations, and interaction patterns

**DOI:** 10.1186/s12870-024-04969-x

**Published:** 2024-04-18

**Authors:** Zhi Zou, Yujiao Zheng, Lili Chang, Liangping Zou, Li Zhang, Yi Min, Yongguo Zhao

**Affiliations:** 1 National Key Laboratory for Tropical Crop Breeding/Hainan Key Laboratory for Biosafety Monitoring and Molecular Breeding in Off-Season Reproduction Regions, Institute of Tropical Biosciences and Biotechnology/Sanya Research Institute of Chinese Academy of Tropical Agricultural Sciences, Haikou, Hainan 571101 P. R. China; 2 Hubei Provincial Key Laboratory for Protection and Application of Special Plants in Wuling Area of China, College of Life Science, South-Central Minzu University, Wuhan, Hubei 430074 P. R. China; 3 College of Biology and Food Engineering, Guangdong University of Petrochemical Technology, Maoming, Guangdong 525000 P. R. China; 4 Hainan University, Haikou, Hainan 570228 P. R. China

**Keywords:** Tigernut, Tonoplast intrinsic protein, Synteny analysis, Expression divergence, Subcellular localization, Protein interaction

## Abstract

**Background:**

Tonoplast intrinsic proteins (TIPs), which typically mediate water transport across vacuolar membranes, play an essential role in plant growth, development, and stress responses. However, their characterization in tigernut (*Cyperus esculentus* L.), an oil-bearing tuber plant of the Cyperaceae family, is still in the infancy.

**Results:**

In this study, a first genome-wide characterization of the *TIP* subfamily was conducted in tigernut, resulting in ten members representing five previously defined phylogenetic groups, i.e., TIP1–5. Although the gene amounts are equal to that present in two model plants Arabidopsis and rice, the group composition and/or evolution pattern were shown to be different. Except for *CeTIP1;3* that has no counterpart in both Arabidopsis and rice, complex orthologous relationships of 1:1, 1:2, 1:3, 2:1, and 2:2 were observed. Expansion of the *CeTIP* subfamily was contributed by whole-genome duplication (WGD), transposed, and dispersed duplications. In contrast to the recent WGD-derivation of *CeTIP3;1*/*-3;2*, synteny analyses indicated that TIP4 and − 5 are old WGD repeats of TIP2, appearing sometime before monocot-eudicot divergence. Expression analysis revealed that *CeTIP* genes exhibit diverse expression profiles and are subjected to developmental and diurnal fluctuation regulation. Moreover, when transiently overexpressed in tobacco leaves, CeTIP1;1 was shown to locate in the vacuolar membrane and function in homo/heteromultimer, whereas CeTIP2;1 is located in the cell membrane and only function in heteromultimer. Interestingly, CeTIP1;1 could mediate the tonoplast-localization of CeTIP2;1 via protein interaction, implying complex regulatory patterns.

**Conclusions:**

Our findings provide a global view of *CeTIP* genes, which provide valuable information for further functional analysis and genetic improvement through manipulating key members in tigernut.

**Supplementary Information:**

The online version contains supplementary material available at 10.1186/s12870-024-04969-x.

## Background

Aquaporins (AQPs), which belong to the ancient major intrinsic protein (MIP) superfamily, constitute a family of small integral membrane proteins facilitating the fast and passive movement of water and other small solutes [1]. In higher plants, AQPs could be classified into five subfamilies on the basis of sequence similarity, i.e., plasma membrane intrinsic protein (PIP), tonoplast intrinsic protein (TIP), NOD26-like intrinsic protein (NIP), small basic intrinsic protein (SIP), and X intrinsic protein (XIP) [2–4]. Among them, PIPs and TIPs, which are typically located in cell and vacuolar membranes, respectively, are vital for maintaining proper cytosolic osmolarity in plant cells [5]. In contrast to only two phylogenetic groups present in the PIP subfamily, the TIP subfamily is highly diverse and five groups have been described not only in Arabidopsis (*Arabidopsis thaliana* (L.) Heynh) but also in rice (*Oryza sativa* L.), two model plants for eudicots and monocots, respectively, implying their early origin [6, 7]. Interestingly, despite the presence of ten members in both Arabidopsis and rice, their group distribution is distinct, reflecting the occurrence of three independent whole-genome duplication (WGD) events after monocot-eudicot divergence [8, 9]. Functional analyses of *TIP* genes have been performed in a wide range of plant species, e.g., Arabidopsis, maize (*Zea mays* L.), rice, wheat (*Triticum aestivum* L.), and tobacco (*Nicotiana tabacum* L.) [10–17]. In addition to water, TIPs have also been shown to transport urea, ammonia (NH_3_), hydrogen peroxide (H_2_O_2_), and glycerol, corresponding to diverse aromatic/arginine (ar/R) selectivity filters that determine the substrate specificity [10–17]. The crystal structure of Arabidopsis AtTIP2;1, a water and ammonia transporter, was resolved in 2016 [18]. Compared with other AQPs such as SoPIP2;1 of spinach (*Spinacia oleracea* L.) [19], the side chain of the conserved R residue in AtTIP2;1 is pushed to the side of the pore by an H residue located in loop C (LC), appearing as a fifth residue of an extended selectivity filter and the relatively wide pore and the polar nature of the selectivity filter allowing the ammonia permeability [18].

Tigernut (*Cyperus esculentus* L. var. *sativus*) is an herbaceous perennial C_4_ plant of the Cyperaceae family within the Poales order, which also includes the well-known Poaceae family [20–23]. Although most likely originating from the Mediterranean and Southwest Asia, tigernut is now widely distributed in tropical, subtropical, temperate, and even cold zones [21, 24, 25]. It has emerged as a novel oil crop that uniquely accumulates up to 35% of oil in underground tubers [26–28]. Despite its strong anti-adversity and wide adaptability [24], the mechanism underlying is poorly understood. Given essential roles of TIPs in plant growth, development, and stress responses [17], in the current study, we took advantage of recently available genome and transcriptome data [29, 30] to identify the complete set of *TIP* subfamily genes. Their gene localizations, gene structures, sequence characteristics, and evolutionary relationships were comprehensively examined and compared with that of Arabidopsis and rice, which have extensively studied [7, 10, 14]. Moreover, gene expression profiles, protein subcellular localizations, and interaction patterns were also investigated, which facilitate further functional analysis and application.

## Results

### Identification and classification of *TIP* genes in tigernut

**Fig. 1:**
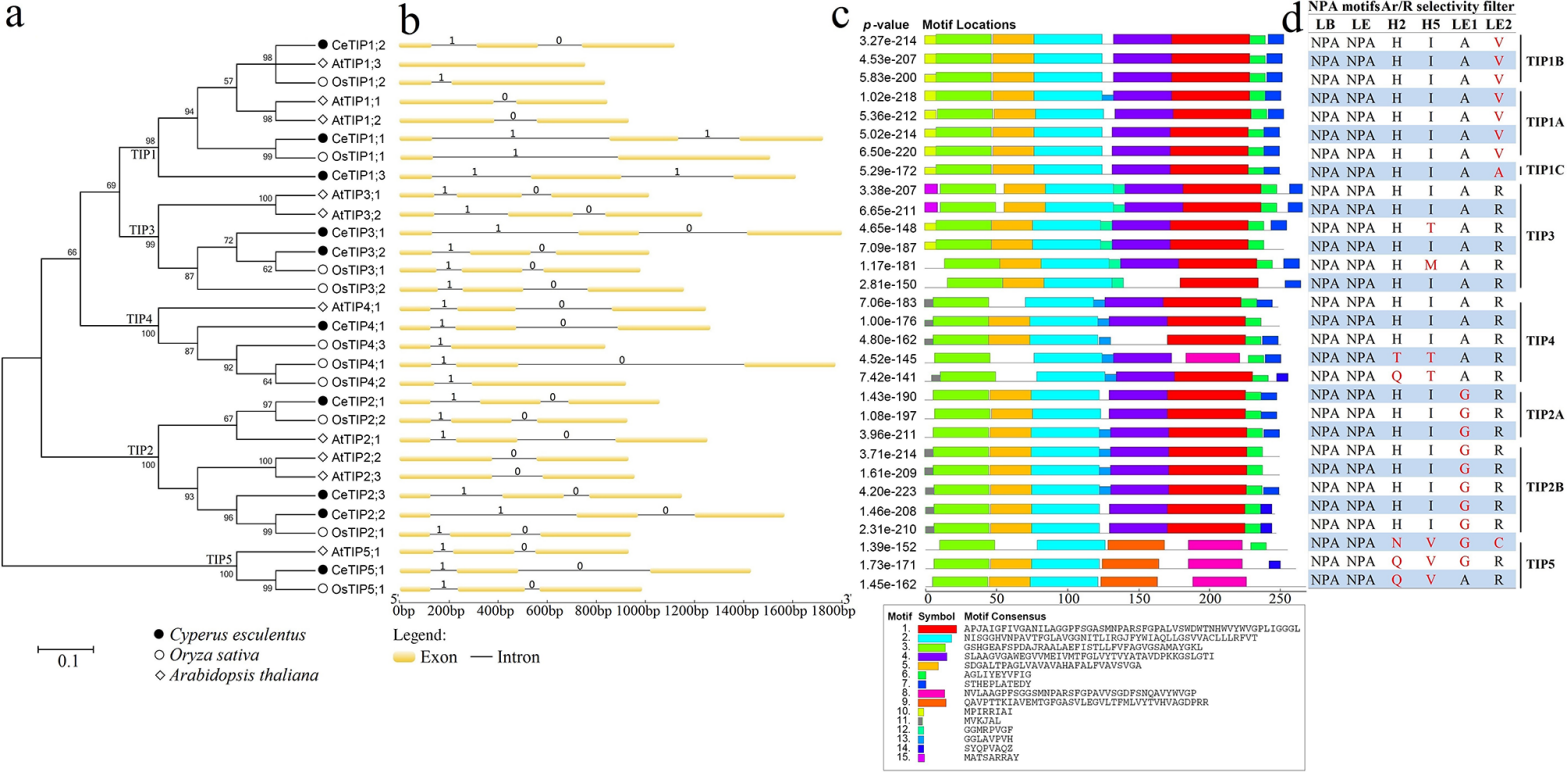
Phylogenetic analysis and structural features of the *TIP* subfamily in tigernut, rice and Arabidopsis. **a** Shown is an unrooted phylogenetic tree resulting from full-length TIPs with MEGA6 (maximum likelihood method and bootstrap of 1,000 replicates), where the distance scale denotes the number of amino acid substitutions per site and the name of each group is indicated next to the corresponding clade. **b** Shown are the exon-intron structures. 0 and 1 indicate intron phases. **c** Shown is the distribution of conserved motifs among TIPs, where different motifs are represented by different color blocks as indicated and the same color block in different proteins indicates a certain motif. **d** Shown are the conserved residues such as dual NPA motifs and Ar/R selectivity filter. Unusual residues are marked in red. (*At Arabidopsis thaliana*, Ar/R aromatic/arginine, *bp* base pair, *Ce Cyperus esculentus*, *H2* transmembrane helix 2, *H5* transmembrane helix 5, *LB* loop B, *LE* loop E, *NPA* Asn-Pro-Ala, *Os Oryza sativa*, *TIP* tonoplast intrinsic protein)

As shown in Table 1, a total of ten *TIP* genes were identified from the tigernut genome and all of them were predicted by the genome annotation [30] and detected in the full-length transcriptome [28], supporting their expression. To uncover their evolutionary relationships, an unrooted phylogenetic tree from deduced polypeptides of *CeTIP*, *OsTIP*, and *AtTIP* genes was constructed as shown in Fig. 1a, which clusters ten *CeTIP* genes into five main groups, i.e., three TIP1s, three TIP2s, two TIP3s, one TIP4, and one TIP5. Among them, TIP1 and − 2 could be divided into several subgroups. Surprisingly, the group distribution in tigernut appears be more similar to Arabidopsis but distinct from rice, though tigernut and rice share a closer biological relationship and both are monocots belonging to Poales [20].

**Table 1:** List of ten *TIP* genes identified in tigernut. (*AA* amino acid, *AI* aliphatic index, *At Arabidopsis thaliana*, Scf scaffold, *GRAVY* grand average of hydropathicity, *kDa* kilodalton, *MIP* major intrinsic protein, *MW* molecular weight, *Os Oryza sativa*, *TIP* tonoplast intrinsic protein, *TM* transmembrane helix, *WGD* whole-genome duplication)

Gene name	Locus	Position	AA	MW (kDa)	pI	GRAVY	AI	MIP	TM	Ortholog	
										Rice	Arabidopsis
*CeTIP1;1*	CESC_05136	Scf16:2974020.2975745(+)	251	25.75	6.01	0.831	107.77	17.233	6	*OsTIP1;1*	*AtTIP1;1, -1;2*
*CeTIP1;2*	CESC_21713	Scf31:3827216.3828338(+)	253	26.03	5.71	0.808	109.57	14.234	6	*OsTIP1;2*	*AtTIP1;3*
*CeTIP1;3*	CESC_14421	Scf2:3957060.3958674(-)	251	25.47	7.00	0.818	107.81	15.233	6	-	-
*CeTIP2;1*	CESC_09964	Scf27:1400602.1401661(+)	248	24.73	5.09	0.948	114.60	14.231	6	*OsTIP2;2*	*AtTIP2;1*
*CeTIP2;2*	CESC_15905	Scf54:4665053.4666621(-)	247	24.80	5.80	1.005	114.25	14.231	6	*OsTIP2;1*	*AtTIP2;2, -2;3*
*CeTIP2;3*	CESC_04647	Scf24:3657482.3658632(-)	251	25.33	5.46	0.944	115.90	14.232	6	*OsTIP2;1*	*AtTIP2;2, -2;3*
*CeTIP3;1*	CESC_17492	Scf16:440841.442643(+)	255	27.12	6.75	0.639	114.16	16.233	6	*OsTIP3;1, -3;2*	*AtTIP3;1, -3;2*
*CeTIP3;2*	CESC_21938	Scf54:3113942.3114971(+)	253	26.69	9.66	0.537	103.12	17.233	6	*OsTIP3;1, -3;2*	*AtTIP3;1, -3;2*
*CeTIP4;1*	CESC_11872	Scf40:5230058.5231324(-)	250	25.57	6.02	0.884	119.40	12.231	6	*OsTIP4;1, -4;2, -4;3*	*AtTIP4;1*
*CeTIP5;1*	CESC_15904	Scf54:4662060.4663490(-)	262	26.82	8.43	0.708	97.63	15.235	6	*OsTIP5;1*	*AtTIP5;1*

The coding sequence (CDS) length of *CeTIP* genes varies from 744 to 789 base pairs (bp), putatively encoding 247–262 amino acids (AA) with the molecular weight (MW) of 24.73–27.12 kilodalton (kDa) (Table 1), which is comparative to that observed in rice (i.e. 248–269 AA; 24.88–27.55 kDa) and Arabidopsis (i.e. 249–268 AA; 25.03–28.31 kDa) (Additional file 1). Whereas most members are acidic with the theoretical pI value of less than 7.00 (i.e. 5.09–6.75), CeTIP3;2 and − 5 appear to be basic (Table 1). It’s worth noting that OsTIP3;1, OsTIP3;2, OsTIP5;1, AtTIP3;1, and AtTIP5;1 were also shown to be basic (Additional file 1). Without any exception, all CeTIPs exhibit the amphipathic property with high aliphatic index (AI) values (i.e. 97.63–119.40) and grand average of hydropathicity (GRAVY) values of more than 0 (i.e. 0.537–1.005) (Table 1), which is consistent with that observed in rice and Arabidopsis (Additional file 1). Sequence similarities between CeTIP proteins vary from 51.60 to 92.43%, where 73.73–85.83%, 82.54–92.43%, and 75.39% were found within three multi-member groups, i.e., TIP1, -2, and − 3. Compared with other groups, TIP5 is relatively distinct, only sharing 51.60–59.55% sequence similarities (Additional file 2). Interestingly, except for CeTIP1;3, all other members have one to three orthologs in rice and Arabidopsis (Table 1), implying lineage or species-specific evolution after their divergence. Significantly, despite the presence of two TIP3s in all three species tested, 2:2 but not 1:1 orthologous relationships were observed (Table 1), implying their birth after speciation.

### Gene localization and synteny analysis of *CeTIP* genes

**Fig. 2:**
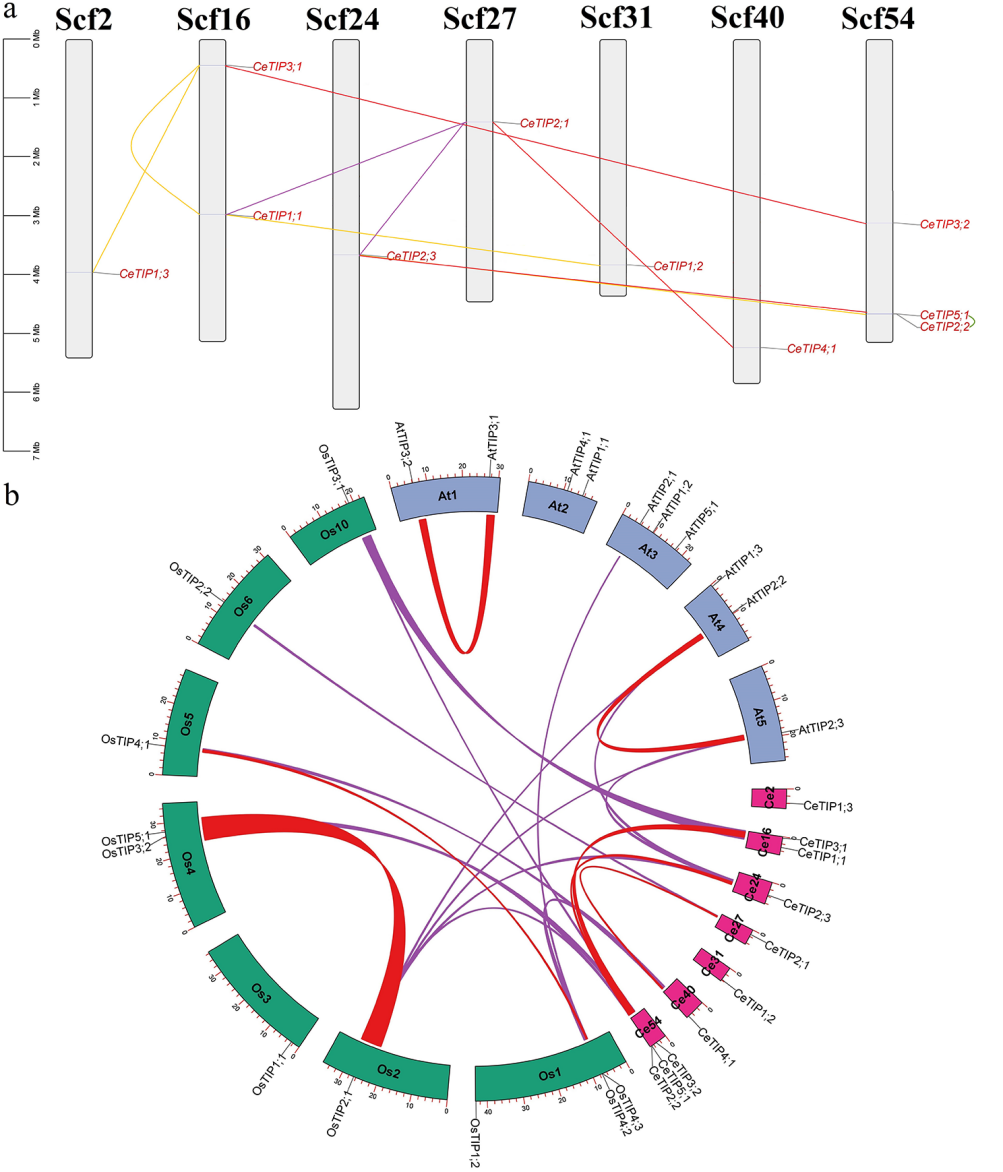
Duplication events of *CeTIP* genes and synteny analysis within and between tigernut, rice, and Arabidopsis. **a** Duplication events detected in tigernut. Serial numbers are indicated at the top of each scaffold, and the scale is in Mb. Duplicate pairs identified in this study are connected using lines in different colors, i.e., tandem (shown in green), transposed (shown in purple), dispersed (shown in gold), and WGD (shown in red). **b** Synteny analysis within and between tigernut, rice, and **A**rabidopsis. Shown are *TIP-*encoding chromosomes/scaffolds (tigernut, pink; rice, dark green; Arabidopsis, bluish-grey) and only syntenic blocks that contain *TIP* genes are marked (intra-species, red; inter-species, purple). (*At Arabidopsis thaliana*, *Ce Cyperus esculentus*, *Mb* megabase, *Os Oryza sativa*, *Scf* scaffold, *TIP* tonoplast intrinsic protein, *WGD* whole-genome duplication)

To learn more about species-specific evolution patterns, gene localization and synteny analysis were further conducted. As shown in Fig. 2a, gene localization showed that ten *CeTIP* genes are unevenly distributed over seven scaffolds (Scfs), where 71.43% of them possess a single gene, one contains two, and Scf54 includes the maximum of three, i.e., *CeTIP3;2*, *-5;1*, and *− 2;2*. Interestingly, *CeTIP5;1* and *− 2;2*, which share 59.55% sequence similarity at the protein level (Additional file 2), could be defined as tandem repeats spacing only 1,254 bp, whereas *CeTIP1;1* and *− 2;3* were characterized as transposed repeats of *CeTIP2;1* (Fig. 2a). According to synteny analysis performed within tigernut, *CeTIP2;1*/-*4;1*, *CeTIP2;3*/-*5;1*, and *CeTIP3;1*/-*3;2*, which respectively exhibit 70.92%, 57.04%, and 75.39% sequence similarities at the protein level (Additional file 2), were shown to locate within syntenic blocks and thus were defined as WGD repeats (Fig. 2b). Additionally, *CeTIP2;2* and − *2;3*, which share 92.43% sequence similarity at the protein level (Additional file 2), were characterized as dispersed repeats, and *CeTIP1;2*, *-1;3*, and *− 3;1* were also defined as dispersed repeats of *CeTIP1;1* (Fig. 2a). By contrast, relatively less WGD repeats were identified in rice (i.e. *OsTIP2;1*/*-5;1* and *OsTIP4;1*/*-4;3*) and Arabidopsis (i.e. *AtTIP2;2*/*2;3* and *AtTIP3;1*/-*3;2*) (Fig. 2b), though both of them experienced three rounds of WGDs after monocot-eudicot divergence [8, 9]. According to inter-species syntenic analysis, six *CeTIP* genes were shown to have syntelogs in rice, including 1:1, 1:2, and 2:1, in striking contrast to only two found in Arabidopsis (i.e. *AtTIP2;2* and *AtTIP2;3* vs. *CeTIP2;3*) (Fig. 2b), reflecting lineage or species-specific duplication events followed by chromosomal rearrangement and gene transposition [31, 32]. These results together with BRH (best reciprocal hit)-based orthologous analysis suggest that (1) TIP2 is more likely to give rise to TIP4 and − 5 via WGDs, occurred sometime before monocot-eudicot divergence; (2) TIP2 had also diverged into two subgroup (i.e. TIP2A and -B), occurred sometime before monocot-eudicot split; (3) similar to the α WGD-derivation of *AtTIP3;1*/*-3;2*, independent expansion of TIP3 may occur sometime after the split of Cyperaceae and Poaceae, e.g., *CeTIP3;1*/*-3;2*, and *OsTIP3;1*/*-3;2*; (4) Cyperaceae-specific *CeTIP1;3* may be generated sometime after Cyperaceae-Poaceae split since its orthologs were also not found in other monocots with genome sequences available in Phytozome; (5) like TIP3, -4 expansion observed in rice was derived from tandem duplication and the Poaceae-specific ρ WGD, and their orthologs were widely found in Poaceae species (Additional file 3).

### Analyses of exon-intron organization and conserved motifs

To gain insights into structural divergence of *CeTIP* genes, their gene structures and conserved motifs of deduced proteins were further investigated and compared with that of rice and Arabidopsis. As shown in Fig. 1b, all *CeTIP* genes possess two introns and three exons, by contrast, nearly half of *TIP* genes in rice and Arabidopsis harbor a single intron and *AtTIP1;3* is intronless. Genes with less than two introns were found in TIP1 and − 4 of rice and TIP1 and − 2 of Arabidopsis, respectively (Fig. 1b). Without any exception, all *CeTIP* genes feature the typical GT/AG splice junction. Generally, intron 1 is located near the end of transmembrane helix 1 (TM1), whereas intron 2 is located just before the A/R/E/S/P/G/H codon of the LC. Whereas intron 1 is the phase 1 intron that is located within codons, intron 2 is usually the phase 0 intron that is located between codons (Fig. 1b). Nevertheless, intron 2 of both *CeTIP1;1* and *− 1;3* exhibits the phase 1 intron (Fig. 1b), which was shown to locate at the C-terminus of TM4 and the loop D (LD), respectively (Additional file 4).

Fifteen motifs identified using MEME are shown in Fig. 1c. Only Motifs 2 and 3, which are characterized as TM1 and LB-TM3 (Additional file 4), are shared by all 30 TIPs examined. Motifs 1 and 4–7 are also broadly present: Motif 1, which is characterized as TM5-LE-TM6, is replaced by Motif 8 in OsTIP4;1 and all TIP5s; Motif 4, which is characterized as LC-TM4-LD, is absent from OsTIP3;2 and OsTIP4;3 and replaced by Motif 9 in all TIP5s; Motif 5, which is characterized as LA-TM2, is absent from AtTIP4;1, OsTIP4;1, OsTIP4;2, and AtTIP5;1; Motif 5, which is characterized as a part of TM6, is absent from CeTIP5;1 and OsTIP5;1; Motif 5, which is located at the C-terminus, is absent from CeTIP3;2, CeTIP4;1, AtTIP2;2, AtTIP2;3, AtTIP5;1, and OsTIP5;1 and replaced by Motif 14 in CeTIP2;2, OsTIP2;1, OsTIP4;2, and CeTIP5;1. Motifs 10, 11, and 15, which are located at the N-terminus, are mainly present in TIP1s, TIP2s/TIP4s, and AtTIP3s, respectively. Motif 12, which is located at LC, is restricted to TIP3 and replaced by Motif 13 in TIP4s, AtTIP2;1, AtTIP2;2, AtTIP2;3, CeTIP2;3, and AtTIP1;1 (Fig. 1c and Additional file 4). It’s worth noting that the motif composition in TIP5s is obviously different from other groups, reflecting their sequence divergence. Nevertheless, all of them harbor one conserved MIP domain that includes six typical TMs (TM1–6), two half helixes (HB and HE), and two typical NPA motifs as observed in SoPIP2;1 [19], though the ar/R selectivity filter is variable (Fig. 1d). Among them, CeTIP1;3 exhibits the H-I-A-A variant in contrast to the H-I-A-V filter widely present in TIP1. TIP3 and − 4 usually feature the H-I-A-R filter, however, CeTIP3;1 exhibits the H-T-A-R variant that is not observed in other members. Whereas all TIP2s feature the H-I-G-R filter, CeTIP5;1 exhibits the Q-V-G-R filter that is different from that of both OsTIP5;1 (Q-V-A-R) and AtTIP5;1 (N-V-G-C). An L residue, corresponding to the L^197^ of SoPIP2;1 that was proven to be involved in gating [19], is only present in CeTIP4;1, OsTIP4;3, and OsTIP5;1. In most cases, this position is replaced by an I residue. An H residue, corresponding to the H^197^ of AtPIP2;2 that was reported to undergo gating by cytosolic pH via protonation [33], is found in CeTIP3;1 and OsTIP3;1. Interestingly, an H residue, corresponding to the H^131^ in the LC of AtTIP2;1 and VvTIP2;1 that is essential for ammonia permeability [18] and involved in gating by cytosolic pH [34], was found not only in TIP2s but also TIP4s, implying their similar functions.

### Global expression profiles of *CeTIP* genes

**Fig. 3:**
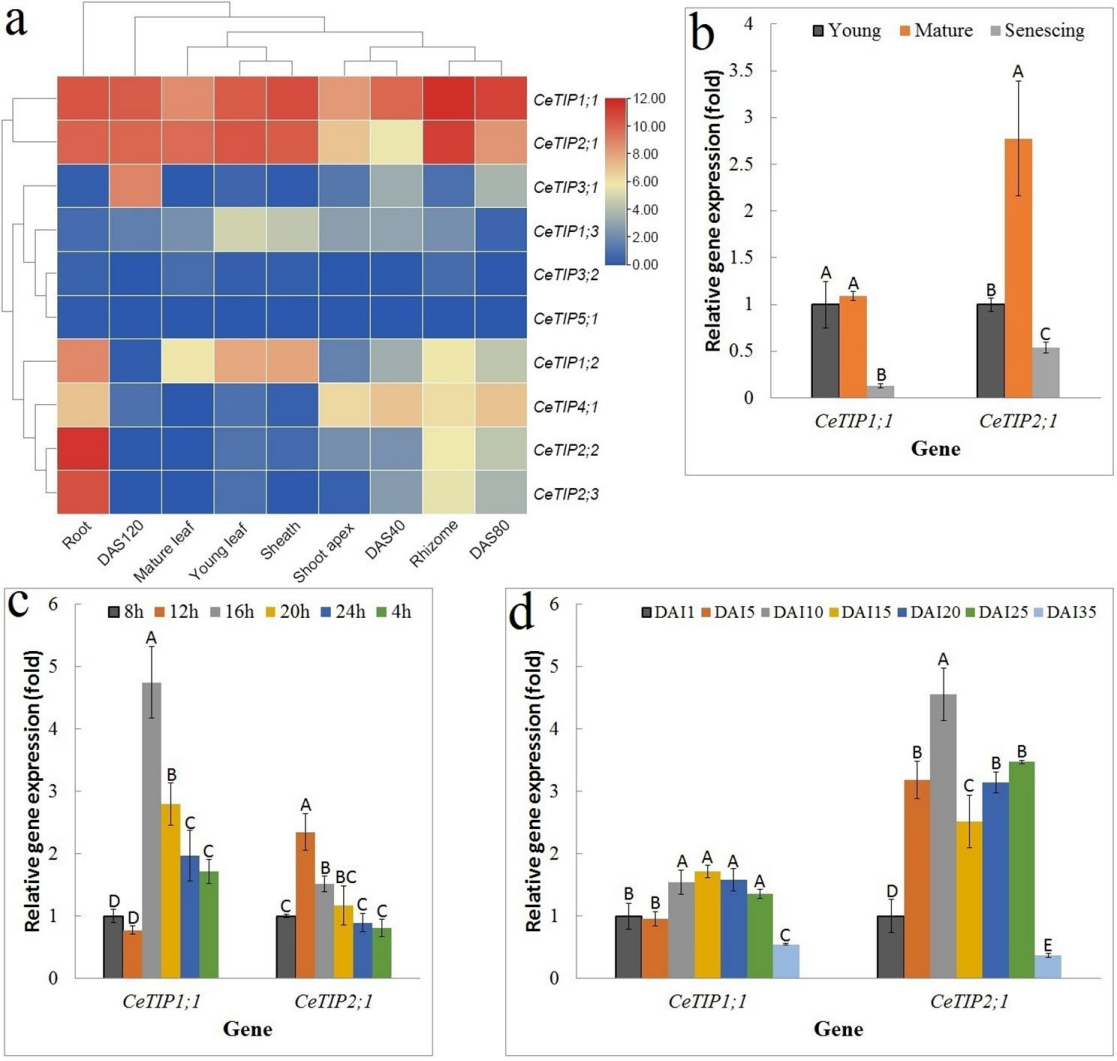
Expression profiles of *CeTIP* genes. **a** Tissue-specific expression profiles of *CeTIP* genes. DAS40, DAS80, and DAS120 represent tubers of 40, 80 and 120 DAS. Color scale represents FKPM normalized log_2_ transformed counts where blue and red indicate low and high expression, respectively. **b** Expression profiles of *CeTIP1;1* and *− 2;1* during leaf development. **c** Diurnal fluctuation expression patterns of *CeTIP1;1* and *− 2;1* in mature leaf. **d** Expression profiles of *CeTIP1;1* and *− 2;1* during tuber development. For qRT-PCR analysis, *CeUCE2* and *CeTIP41* were used as two reference genes. Bars and error bars indicate the mean ± SD (*n* = 3) and uppercase letters indicate difference significance tested following Duncan’s one-way multiple-range post hoc ANOVA (*P* < 0.01). (*Ce Cyperus esculentus*, *DAI* days after tuber initiation, *DAS* days after sowing, *TIP* tonoplast intrinsic protein)

To reveal the global expression profiles of *CeTIP* genes, RNA-seq data for nine tissues/developmental stages with three biological repeats each were first examined, i.e., two stages of developmental leaf (young and mature), sheath of mature leaf, root, rhizome, shoot apex, and three stages of developmental tuber (40, 80, and 120 days after sowing (DAS)). As shown in Fig. 3a, the total *CeTIP* transcripts were most abundant in roots, followed by rhizomes, moderate in sheaths, young leaves, and tubers of 120 and 80 DAS, and relatively low in mature leaves, tubers of 40 DAS, and shoot apexes. Interestingly, except for roots and tubers of 40 DAS, *CeTIP1;1* and *− 2;1* represent two dominant members in all other samples examined, contributing 82.46–95.72% of total *TIP* transcripts. In roots, *CeTIP2;2* and *− 2;3* were shown to express more than *− 1;1* and *− 2;1*, and these four members constituted more than 91.89% of total *TIP* transcripts. Additionally, *CeTIP1;2* and *− 4;1* were also abundant in roots, whose transcripts were shown to be comparative or considerably more than that of most other samples. In shoot apexes, *CeTIP4;1* represented the third most expressed member, contributing 15.02% of total *TIP* transcripts. By contrast, this gene was rarely expressed in leaves and sheaths. Interestingly, *CeTIP3;1* appeared to be tuber-specific and highly abundant in tubers of 120 DAS, whereas *CeTIP3;2* was lowly expressed in all samples examined.

### Expression profiles of *CeTIP* genes during leaf development and diurnal fluctuation

As shown in Fig. 3a, most *CeTIP* genes were significantly down-regulated in mature leaves relative to young leaves, including two dominant genes *CeTIP1;1* and *− 2;1*. However, this is the case under a natural condition. To provide a more accurate determination, plants grown in a greenhouse as described before [23] were adopted and three representative stages of developmental leaf (i.e. young, mature, and senescing) were subjected to qRT-PCR analysis. As shown in Fig. 3b, both *CeTIP1;1* and *− 2;1* exhibited a bell-shaped expression pattern, peaking in mature leaves, though the difference of *CeTIP1;1* between young and mature leaves was not significant.

Diurnal fluctuation expression patterns of *CeTIP1;1* and *− 2;1* in mature leaves were further examined. As shown in Fig. 3c, since the onset of light at 8 h, their transcripts usually increased along with the extension of the light time, peaking at 16 h and 12 h, respectively, though a slight drop was observed at 12 h for *CeTIP1;1*. Whereas *CeTIP2;1* was expressed more in light (i.e. 8 h, 12 h, 16 h, and 20 h) relative to dark (i.e. 24 h and 4 h), the transcripts of *CeTIP1;1* at 8 h and 12 h were significantly lower than that in dark (Fig. 3c).

### Expression profiles of *CeTIP* genes during tuber development

The oil-bearing tubers are derived from rhizomes and the development process includes three main stages, i.e., initiation, swelling, and maturation, spanning approximately 35 d [22]. In contrast to gradual increase of dry weight and oil during tuber development, the water content usually maintains up to 85.0% until a significant drop to less than 45.0% at the stage of maturation [22, 26, 28], implying a key role of water for tuber development and metabolism. Among three swelling stages profiled, except for *CeTIP1;3* that peaked at 40 DAS, transcripts of *CeTIP2;1* and *− 3;1* were shown to gradually increase, whereas five genes (i.e. *CeTIP1;1*, *-1;2*, *-2;2*, *-2;3*, and *− 4;1*) exhibited an apparent unimodal expression pattern, peaking at 80 DAS (Fig. 3a).

For qRT-PCR analysis, seven stages were examined, i.e., 1, 5, 10, 15, 20, 25, and 35 days after tuber initiation (DAI), representing initiation, five stages of swelling, and maturation as described before [22]. As shown in Fig. 3d, *CeTIP1;1* exhibited a nearly bell-shaped expression pattern, peaking at 15 DAI, which is similar to the transcriptome profiling. By contrast, a bimodal expression pattern was observed for *CeTIP2;1*, peaking at 10 and 25 DAI, respectively. It’s worth noting that, for both genes, transcripts at the maturation stage were significantly lower than any other stage (Fig. 3d), corresponding to relatively low water content at this stage [22].

### Subcellular localization analysis

**Fig. 4:**
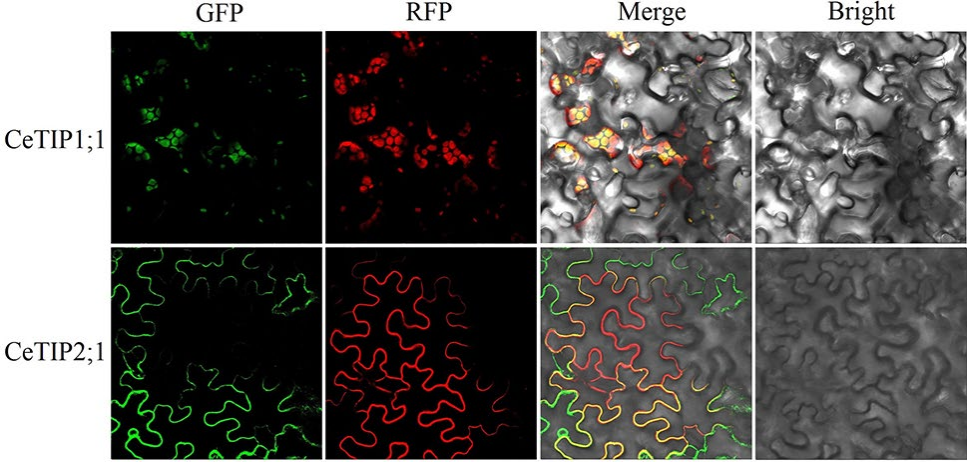
Subcellular localization analysis of CeTIP1;1 and − 2;1 in tobacco leaves. AtTIP1;1-RFP and HbPIP2;3-RFP were used as markers for tonoplast and plasma membrane, respectively. (*Ce Cyperus esculentus*, *TIP* tonoplast intrinsic protein)

According to bioinformatic prediction using Plant-mPLoc, all CeTIP proteins were shown to localize to the tonoplast. To verify the result, two dominant members *CeTIP1;1* and *− 2;1* were selected for experimental confirmation. As shown in Fig. 4, green fluorescence signals of CeTIP1;1-GFP were widely detected in vacuoles of tobacco leaves, highly coinciding with the tonoplast marker AtTIP1;1-RFP [10]. By contrast, to our surprise, fluorescence signals of CeTIP2;1-GFP were only found in cell membranes, coinciding with the plasma membrane marker HbPIP2;3-RFP [35].

### Protein interaction patterns

**Fig. 5:**
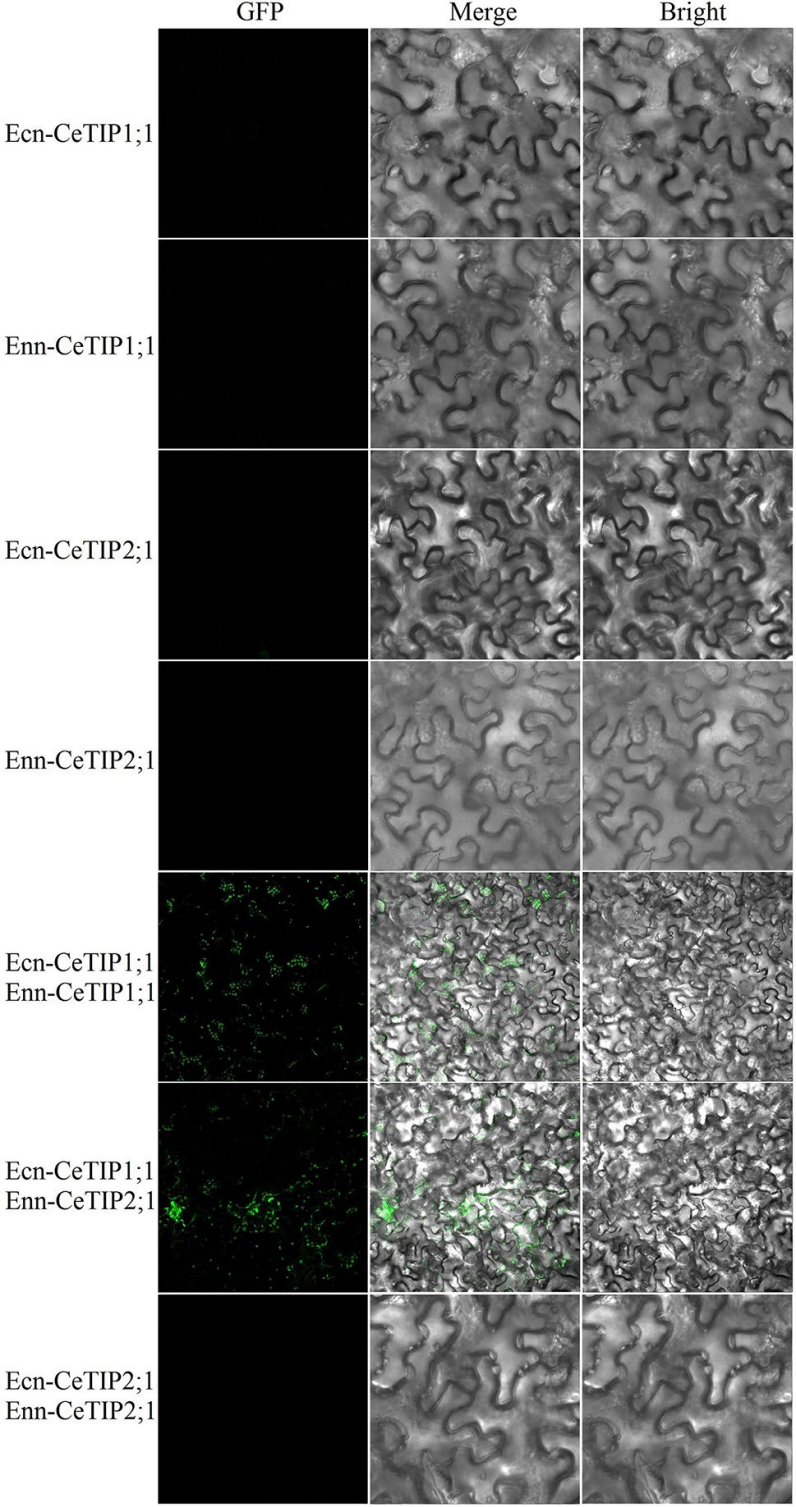
BiFC-based protein interaction of CeTIP1;1 and CeTIP2;1 in tobacco leaves. Tobacco leaves transformed with *p*NC-BiFC-Ecn-*CeTIP1;1*, *p*NC-BiFC-Enn-*CeTIP1;1*, *p*NC-BiFC-Ecn-*CeTIP2;1*, or *p*NC-BiFC-Ecn-*CeTIP2;1* were used as negative controls. (*BiFC* bimolecular fluorescence complementation, *Ce Cyperus esculentus*, *TIP* tonoplast intrinsic protein)

Constitutive expression and high abundance of *CeTIP1;1* and *− 2;1* (Fig. 3a) suggest that their encoded proteins may interact with each other. To confirm the hypothesis, bimolecular fluorescence complementation (BiFC) analysis was conducted. As shown in Fig. 5, in contrast to no signals were detected for four negative controls, green fluorescence signals were found in vacuoles of tobacco leaves co-transformed with Ecn-CeTIP1;1 and Enn-CeTIP2;1, supporting their interaction. Moreover, homologous interaction was also observed for CeTIP1;1 but not − 2;1. These results imply that CeTIP1;1 is more likely to function in homo and heteromultimer, whereas CeTIP2;1 functions in heteromultimer (Fig. 5).

## Discussion

### The tigernut genome encodes ten *TIP* genes with two introns

Since the first *TIP* gene (i.e. *AtTIP1;1* or*γ-TIP1*) was characterized in Arabidopsis [10], its homologs have been identified in a high number of plant species, varying from eight to more than 23 members in soybean (*Glycine max* (L.) Merr.) [10, 16, 17, 36, 37]. In the present study, a number of ten *TIP* genes that is equal to that of Arabidopsis were identified from tigernut, an oil-bearing tuber plant of the Cyperaceae family, which represents a sister family to Poaceae within the Poales order [20]. The *CeTIP* gene amounts are comparative to or relatively less than 9–15 members present in most Poaceae plants, e.g., rice, barley (*Hordeum vulgare* L.), *Brachypodium distachyon* (L.) P. Beauv., foxtail millet (*Setaria italic* (L.) P. Beauv.), green foxtail (*S. viridis* (L.) P. Beauv.), sorghum (*Sorghum bicolor* (L.) Moench), and maize [7, 16, 38–40], Additional file 3. Nevertheless, all these genes could be assigned into five phylogenetic groups as defined in Arabidopsis, i.e., TIP1–5 [6]. Moreover, in contrast to a high number of *TIP* genes present in Arabidopsis and rice harboring no or a single intron, all *CeTIP* genes feature two introns. Constant positions of two introns in most *TIP* genes support independent loss of one or both introns, whereas varied intron phase and locations of intron 2 in *CeTIP1;1* and *− 1;3* imply the initial loss followed by gain of a new intron. Similar phenomenon was also reported for *AtPIP2;4* [6].

### Expansion of the *CeTIP* subfamily was contributed by WGD, transposed, and dispersed duplications

Gene duplication, which may originate from WGD, tandem, proximal, dispersed, and transposed duplications, is a major mechanism for acquiring new genes [41]. Increasing evidences showed that WGDs are widespread and play an important role in the diversification of angiosperms [42, 43]. For example, after monocot-eudicot split, the model eudicot Arabidopsis experienced three rounds of WGDs named γ, β, and α, respectively [8], whereas the model monocot rice experienced three WGDs known as τ, σ, and ρ, respectively [9]. The ρ WGD identified in rice is Poaceae-specific, whereas τ and σ WGDs are shared by Cyperaceae plants, which may also experience at least one recent WGD [22, 23, 44–46]. Wide presence of five phylogenetic groups in both monocots and eudicots [3, 4, 16, 36, 40, 47–49, Additional file 3] support their early diversification. Interestingly, our data from synteny analyses provides the first evidence that both TIP4 and − 5 were derived from TIP2 via WGDs, occurred sometime before monocot-eudicot divergence. As for TIP5, *CeTIP2;3*, *CeTIP5;1*, *OsTIP2;1*, *OsTIP5;1*, *AtTIP2;2*, and *AtTIP2;3* are still located within syntenic blocks, whereas for TIP4, *CeTIP2;1*, *CeTIP4;1*, *OsTIP2;2*, *OsTIP4;1*, *OsTIP4;3*, and *AtTIP2;1* remain within syntenic blocks (Fig. 2b). Considering the distinct relationship of TIP5, if TIP2 is the primitive one, it may first give rise to -5 followed by -4. During later evolution, TIP2 generated − 1 and − 3 via transposed and dispersed duplications, respectively. More recently but before monocot-eudicot divergence, TIP2 and − 1 diverged into two subgroups via a yet unknown mechanism, i.e., TIP2A, -2B, -1 A, and − 1B. Thereby, from an evolutionary perspective, it is more suitable to classify TIP2 and − 1 into four groups. By contrast, expansion of TIP3 in tigernut appears to be lineage-specific, occurred sometime after Cyperaceae and Poaceae split or more likely by the recent WGD described in *Carex littledalei* C. B. Clarke (formerly known as *Kobresia littledalei*) [46], which is similar to the α WGD-derivation of *AtTIP3;1*/-*3;2* (Additional file 1); expansion of TIP4 via both the ρ WGD and tandem duplication observed in rice seems to be Poaceae-specific, though the counterpart of *OsTIP4;2* has lost in barley (Additional file 3). Nevertheless, we are not sure about the exact time and mechanism that *CeTIP1;3* was generated, which is more distinct from *CeTIP1;1* and *− 1;2* but doesn’t seem to have counterparts in other examined species, including seven representative Poaceae species. In fact, besides the evolutionary distance, CeTIP1;3 differs from other TIP1s in both intron phase and ar/R selectivity filter (H-I-A-A vs. H-I-A-V), implying possible functional divergence. Additionally, TIP2 has evolved into the H-I-G-R filter, whereas TIP3 and − 4 usually exhibit the H-I-A-R filter, though some variants were also found (Fig. 1d). Moreover, we also identified two motifs specific to TIP3 (Motif 12) and − 5 (Motif 9), respectively (Fig. 1c).

### *CeTIP* genes exhibit diverse expression profiles and are subjected to development and diurnal fluctuation regulation

Uncovering the gene expression profiles is the first step to address the importance and specific biological functions of a gene in a special tissue or certain developmental stages. According to our transcriptional profiling, *CeTIP1;1* and *− 2;1* were shown to constitutively express and represent two dominant members in most tissues examined, whereas other genes are relatively less expressed or even tissue-specific, e.g., tuber-specific expression of *CeTIP3;1* (Fig. 3a). *CeTIP1;1* has one and two orthologs in rice and Arabidopsis, respectively, i.e., *OsTIP1;1*, *AtTIP1;1*, and *AtTIP1;2*, where *AtTIP1;2* is characterized as a recent transposed repeat of *AtTIP1;1* (Additional file 1). By contrast, *CeTIP2;1* possesses a single ortholog in both rice and Arabidopsis, i.e., *OsTIP2;2* and *AtTIP2;1*, where *CeTIP2;1* and *OsTIP2;2* are still located within syntenic blocks (Fig. 2b), implying a conserved evolution. Interestingly, *OsTIP1;1*, *OsTIP2;2*, *AtTIP1;1*, *AtTIP1;2*, and *AtTIP2;1* were also shown to be highly abundant in most tissues [16, 17, 51, 52], implying their essential roles. Possible functions of *CeTIP1;1* and *− 2;1* could be inferred from their orthologs, which have been proven to transport water, urea, NH_3_, H_2_O_2_, and glycerol [10, 13, 14, 53–57]. Studies showed that *AtTIP1;1* expression is primarily correlated with vacuolation and cell enlargement [50]. Interestingly, despite a long time of evolution, *AtTIP1;1*, *-1;2*, and *− 2;1* are redundantly involved in the emergence of new lateral root primordial [58]. *CeTIP1;2*, a dispersed repeat of *CeTIP1;1* with a high similarity of 85.83%, also exhibits a constitutive expression pattern. However, its transcript levels are considerably lower than that of *CeTIP1;1* and *− 2;1*, and its orthologs in rice and Arabidopsis, i.e., *OsTIP1;2* and *AtTIP1;3*, have been proven to transport water, urea, and glycerol [57, 59]. Interestingly, in Arabidopsis, *AtTIP1;3* is characterized as the pollen-specific and highly abundant *AQP* gene [59]. *CeTIP2;2* and *− 2;3*, two recent dispersed repeats, are preferentially expressed in roots and rhizomes. They have one or two orthologs in rice and Arabidopsis, respectively, i.e., *OsTIP2;1*, *AtTIP2;2*, and *AtTIP2;3*, which have been proven to transport water, NH_3_, and H_2_O_2_ [56, 60]. In rice, *OsTIP2;1* is preferentially expressed in roots [7]. Despite recent origin and remaining within syntenic blocks, *CeTIP3;1* and *− 3;2* exhibit distinct expression pattern, where *CeTIP3;1* is tuber-specific gradually increasing during tuber development and *− 3;2* is lowly expressed in all tissues examined in this study. Their orthologs in Arabidopsis, i.e., *AtTIP3;1* and *− 3;2*, exhibit a strict embryo and endosperm-specific expression pattern [61], in contrast to endosperm-preferential expression of *RcTIP3;1* in castor bean (*Ricinus communis* L.) [3]. Interestingly, *OsTIP3;2* was proven to transport glycerol, which together with seed-specific expression of TIP3s in oilcrops such as canola (*Brassica napus* L.), soybean, peanut (*Arachis hypogaea* L.), and flax (*Linum usitatissimum* L.) imply a putative role in regulating oil content [17]. *CeTIP4;1*, typically expressed in tubers (downregulated during tuber maturation), roots, rhizomes as well as shoot apexes, has one or three orthologs in arabidopsis and rice, respectively, i.e., *AtTIP4;1*, *OsTIP4;1*, *OsTIP4;2*, and *OsTIP4;3*, which have been proven to transport water, urea, and glycerol [14, 54].

Diurnal variation of *AQP* genes has been reported in several species [7, 62]. For example, the transcripts of *OsTIP1;2* and *− 2;1* in roots were shown to exhibit a clear diurnal change with a large amplitude, peaking in light and dropping to the basal level in dark [7]. Similar trend was also observed in this study, and transcripts of both *CeTIP1;1* and *− 2;1* peaked in the daytime, 8 h–4 h after the onset of light, respectively (Fig. 3c).

### Distinct subcellular localizations and interaction patterns of CeTIP1;1 and − 2;1

As the name suggests, TIPs are located mostly in tonoplasts of plant cells, which function in tetramers [5, 10, 18]. Interestingly, *CeTIP1;1* and *− 2;1*, two dominant *CeTIP* genes identified in tigernut, encode proteins with distinct subcellular localizations and interaction patterns. When transiently overexpressed in tobacco leaves, CeTIP1;1 is clearly located in the vacuole membrane, in contrast to CeTIP2;1 that is located in the cell membrane. Moreover, CeTIP2;1 could not interact itself, whereas CeTIP1;1 functions in homo/heteromultimer and could mediate the tonoplast-localization of CeTIP2;1. It’s worth noting that proteomic analysis suggests the presence of AtTIPs in the thylakoid membrane of chloroplast [63], and AtTIP3s were shown to localize in both the plasma membrane and tonoplast of maturing and germinating seeds [61]. Compared with PIPs, only a few studies have been performed to investigate the interaction pattern of TIPs. In Arabidopsis, AtTIP2;1 could not only interact itself but also interact with AtTIP1;2 and − 3;1 [64]. Thereby, the reason for why CeTIP2;1 could not form homomultimer needs to be further studied.

## Conclusions

To our knowledge, this is the first genome-wide characterization of the *TIP* subfamily in tigernut, an oil-bearing tuber plant of the Cyperaceae family. Ten members representing five phylogenetic groups identified in tigernut are equal to that present in two model plants Arabidopsis and rice, however, the group composition and/or evolution pattern are different and complex orthologous relationships were observed. Expansion of the *CeTIP* subfamily was contributed by WGD, transposed, and dispersed duplications. Whereas *CeTIP3;1*/*-3;2* are recent WGD repeats, TIP4 and − 5 were proven to be old WGD repeats of TIP2, appearing sometime before monocot-eudicot divergence. *CeTIP* genes exhibit diverse expression profiles and are subjected to developmental and diurnal fluctuation regulation. Moreover, CeTIP1;1 and − 2;1, two dominant members, exhibit distinct subcellular localizations and interaction patterns. These findings provide valuable information for further functional analysis and genetic improvement through manipulating key members such as *CeTIP1;1* and *− 2;1* in tigernut.

## Materials and methods

### Datasets and identification of *TIP* genes

As shown in Additional file 1, *TIP* genes described in Arabidopsis and rice were obtained from TAIR11 (https://www.arabidopsis.org/) and RGAP7 (http://rice.plantbiology.msu.edu/), respectively. Their deduced proteins were used for TBLASTN (*E*-value, 1e–10) search of tigernut genomic and transcriptome data that were accessed from CNGBdb (https://db.cngb.org/search/assembly/CNA0051961/) and NCBI (https://www.ncbi.nlm.nih.gov/). Gene prediction and exon-intron revision with available mRNAs were conducted as previously described [48]. Briefly, gene structures of candidates were predicted using GeneMark.hmm eukaryotic (http://topaz.gatech.edu/GeneMark/gmhmme.cgi) when no gene models are available, and then manual revision was performed through aligning mRNAs (including full-length transcripts obtained via the Single-Molecule Real-Time (SMRT) sequencing) [29] to the gene sequences. Open reading frames (ORFs) of candidates were predicted using ORF Finder (http://www.bioinformatics.org/sms2/orf_find.html). Presence of the conserved MIP domain in deduced peptides was confirmed by MOTIF Search (https://www.genome.jp/tools/motif/), and physiochemical parameters were calculated using ProtParam (http://web.expasy.org/protparam/). Protein subcellular localization was predicted using Plant-mPLoc (http://www.csbio.sjtu.edu.cn/bioinf/plant-multi/), and gene structure was displayed using GSDS 2.0 (http://gsds.gao-lab.org/).

### Synteny analysis and characterization of orthologs

Synteny analysis was conducted as previously described [28, 65], where duplicate pairs were identified using the all-to-all BLASTP method and syntenic blocks were inferred using MCScanX implemented in TBtools-II [66]: *E*-value, 1e-10; BLAST hits, 5. Orthologs between different species were identified using the BRH method and information from synteny analysis, whereas different modes of gene duplication were identified using the DupGen_finder pipeline [43].

### Sequence alignment, phylogenetic analysis, and characterization of conserved residues/motifs

Multiple sequence alignment was conducted using MUSCLE [67] (gap open: -2.9; gap extend: 0; hydrophobicity multiplier: 1.2; clustering method: UPGMB) and phylogenetic tree was constructed using MEGA6 [68] with the maximum likelihood method (bootstrap method: 1,000 replicates; substitution model: amino acid and Jones-Taylor-Thornton (JTT) model; rates among sites: uniform rates; gaps/missing data treatment: complete deletion; ML heuristic method: Nearest-Neighbor-Interchange (NNI); initial tree for ML: Make initial tree automatically (Default - NJ/BioNJ)). Subclassification of TIPs into groups was done as described before [3, 6]. TMs and conserved residues were identified from the alignment with the structure resolved SoPIP2;1 and AtTIP2;1 [18, 19]. Conserved motifs were identified using MEME (v5.4.1, https://meme-suite.org/tools/meme) with optimized parameters of any number of repetitions, maximum number of 15 motifs, and the width of 6 and 250 residues for each motif.

### Plant materials

For gene cloning and expression analysis, the tigernut variety Reyan3 as described before [23, 28] was used, and plants were grown in a greenhouse under the condition of 25 ± 0.5℃, 60–70% relative humidity, 5,000 lx, and a 12 h light/12 h dark cycle. For a day, the period from 8 a.m to 8 p.m was set to light, and mature leaves with dark green in appearance were sampled every four hours from the onset of light. Three representative stages of developmental leaf, i.e., young, mature, and senescing, were also collected at 8 a.m, where the chlorophyll content of young and senescing leaves are just a half relative to mature leaves. As for representative stages of developmental tuber, fresh tubers at 1, 5, 10, 15, 20, 25, and 35 DAI were collected as described before [22]. All samples with three biological replicates were quickly freezed with liquid nitrogen and stored at -80℃. For subcellular localization and BiFC analyses, *N. benthamiana* L. plants were also grown in the greenhouse as described above.

### Gene expression analysis

Global expression profiles of *CeTIP* genes were investigated using Illumina RNA-seq samples as shown in Additional file 5, which are paired reads with 150 bp. Raw reads in the FASTQ format were obtained using fastq-dump, and quality control was performed using Trimmomatic [69]. Read mapping was performed using HISAT2 (v2.2.1) [70] with default parameters and relative transcript levels were presented in FPKM (Fragments per kilobase of exon per million fragments mapped). Differential expression genes were identified using DESeq2 (v1.22.1) [71] with parameters of “log_2_Fold Change > = 1” and FDR < 0.05. For qRT-PCR analysis, total RNAs were extracted using the RNAprep Pure Plant Kit (Tiangen Biotech Co., Beijing, China); synthesis of the first-strand cDNA from high quality RNAs was carried out using the PrimeScript® RT reagent kit with gDNA Eraser (Takara, Dalian, China); and PCR reaction was carried out as described before [23]. Primers used in this study are shown in Additional file 6, where *CeUCE2* and *CeTIP41* were used as two reference genes. The relative expression level of target genes was normalized using the 2^−ΔΔCt^ method and statistical significance was performed using SPSS Statistics 20, where difference significance was tested following Duncan’s one-way multiple-range post hoc ANOVA.

### Subcellular localization and BiFC

Primers used for plasmid construction are shown in Additional file 6. Full-length transcripts of *CeTIP1;1* and *− 2;1* were first isolated using *CeTIP1;1*F/R and *CeTIP2;1*F/R as primers, respectively. Then, the PCR products were employed as template, and the CDS without the termination codon were cloned to *p*NC-Cam1304-SubC, *p*NC-BiFC-Ecn, and *p*NC-BiFC-Enn using *CeTIP1;1*HF/R and *CeTIP2;1*HF/R as primers. The resulted recombinant plasmids, i.e., *p*NC-Cam1304-*CeTIP1;1*, *p*NC-Cam1304-*CeTIP2;1*, *p*NC-BiFC-Ecn-*CeTIP1;1*, *p*NC-BiFC-Ecn-*CeTIP2;1*, *p*NC-BiFC-Enn-*CeTIP1;1*, and *p*NC-BiFC-Ecn-*CeTIP2;1* were introduced into *Agrobacterium tumefaciens* GV3101 with the helper plasmid *p*Soup-*P19* as described before [72]. *A. tumefaciens*-mediated transformation was carried out as previously described [28, 72], where tobacco leaves of about 4-week-old plants were used as the receptor. About 48 h after infiltration, transformed leaves were processed for confocal laser scanning microscopy imaging (Zeiss LMS880, Germany). For subcellular localization analysis, markers for tonoplast (AtTIP1;1-RFP [10]) and plasma membrane (HbPIP2;3-RFP [35]) were also co-transformed.

### Electronic supplementary material

Below is the link to the electronic supplementary material.

Supplementary Material 1

## Data Availability

The datasets analyzed during the current study are available in the NCBI SRA repository (https://www.ncbi.nlm.nih.gov/sra/) and detailed accession numbers can be found in Additional file 5.
