# Supplementary material for: TIP aquaporins in Cyperus esculentus: genome-wide identification, expression profiles, subcellular localizations, and interaction patterns
Source: BMC Plant Biol. 2024 Apr 18;24:298. doi: 10.1186/s12870-024-04969-x (PMC11025170; doi:10.1186/s12870-024-04969-x)
Supplement: Supplementary file 1 — Supplementary Material 1 [file 12870_2024_4969_MOESM1_ESM.docx]

**Supplementary Information**

The online version contains supplementary material available at https:// doi.org/.

Additional file 1 Detailed information of *TIP* genes present in rice and Arabidopsis.

Additional file 2 Percent similarity between CeTIPs, OsTIPs, and AtTIPs.

Additional file 3 Orthologs for OsTIPs identified in representative Poaceae species with genome sequences available in Phytozome.

Additional file 4 Alignment of CeTIPs, OsTIPs, AtTIPs, and SoPIP2;1.

Additional file 5 Detailed information of transcriptome data used in this study.

Additional file 6 Primers used in this study.
